# 1,1′-{[1,4-Phenyl­enebis(methyl­ene)]bis­(­oxy)bis­(4,1-phenyl­ene)}diethanone

**DOI:** 10.1107/S160053681104565X

**Published:** 2011-11-05

**Authors:** Nassir N. Al-Mohammed, Yatimah Alias, Zanariah Abdullah, Hamid Khaledi

**Affiliations:** a Department of Chemistry, University of Malaya, 50603 Kuala Lumpur, Malaysia

## Abstract

The centroid of the central aromatic ring of the title mol­ecule, C_24_H_22_O_4_, is located on an inversion center. The dihedral angle between the central and terminal benzene rings is 75.00 (7)°. In the crystal, mol­ecules are linked through C—H⋯O hydrogen bonds into chains along [121]. The chains are connected into layers *via* C—H⋯π inter­actions.

## Related literature

For related structures, see: Al-Mohammed *et al.* (2011 ▶); Hu (2010 ▶); Tang *et al.* (2008 ▶).
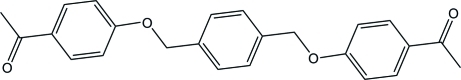


## Experimental

### 

#### Crystal data

C_24_H_22_O_4_
*M* *_r_* = 374.42
Triclinic, 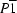

*a* = 8.1286 (12) Å
*b* = 8.1610 (7) Å
*c* = 8.4878 (6) Å
α = 116.164 (5)°
β = 106.328 (7)°
γ = 100.196 (7)°
*V* = 454.41 (8) Å^3^
*Z* = 1
Mo *K*α radiation
μ = 0.09 mm^−1^
*T* = 100 K
0.23 × 0.19 × 0.09 mm

#### Data collection

Bruker APEXII CCD diffractometer
Absorption correction: multi-scan (*SADABS*; Sheldrick, 1996 ▶) *T*_min_ = 0.979, *T*_max_ = 0.992
2732 measured reflections
1654 independent reflections
1472 reflections with *I* > 2σ(*I*)
*R*_int_ = 0.020

#### Refinement

*R*[*F* ^2^ > 2σ(*F* ^2^)] = 0.040
*wR*(*F* ^2^) = 0.112
*S* = 1.07
1654 reflections
128 parameters
H-atom parameters constrained
Δρ_max_ = 0.20 e Å^−3^
Δρ_min_ = −0.34 e Å^−3^

### 

Data collection: *APEX2* (Bruker, 2007 ▶); cell refinement: *SAINT* (Bruker, 2007 ▶); data reduction: *SAINT*; program(s) used to solve structure: *SHELXS97* (Sheldrick, 2008 ▶); program(s) used to refine structure: *SHELXL97* (Sheldrick, 2008 ▶); molecular graphics: *X-SEED* (Barbour, 2001 ▶); software used to prepare material for publication: *SHELXL97* and *publCIF* (Westrip, 2010 ▶).

## Supplementary Material

Crystal structure: contains datablock(s) I, global. DOI: 10.1107/S160053681104565X/pv2469sup1.cif

Structure factors: contains datablock(s) I. DOI: 10.1107/S160053681104565X/pv2469Isup2.hkl

Additional supplementary materials:  crystallographic information; 3D view; checkCIF report

## Figures and Tables

**Table 1:** Hydrogen-bond geometry (Å, °) *Cg*1 is the centroid of the C3–C8 ring.

*D*—H⋯*A*	*D*—H	H⋯*A*	*D*⋯*A*	*D*—H⋯*A*
C11—H11⋯O1^i^	0.95	2.54	3.4362 (18)	158
C12—H12⋯*Cg*1^ii^	0.95	2.61	3.5078 (17)	158

Symmetry codes: (i) 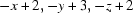; (ii) 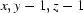.

**Fig. 1.**
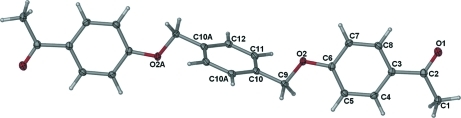
Molecular structure of the title compound with displacement ellipsoids drawn at 50% probability level. Hydrogen atoms are drawn as spheres of arbitrary radius. Symmetry code: A = -x+1, -y+1, -z+1.

**Fig. 2.**
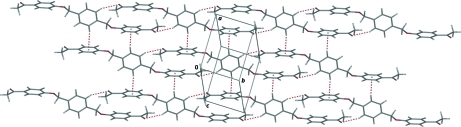
The two-dimensional network formed by C—H···O and C—H···π interactions (dashed lines).

## supplementary crystallographic information

### Comment

We have recently reported the crystal structure of *o*-acetyl isomer of the title compound (Al-Mohammed *et al.*, 2011). Similar to the previous structure, the title molecule shows a centrosymmetric molecular structure with the centroid of the central benzene ring being located on an inversion center. The central and terminal rings make a dihedral angle of 75.00 (7)°. This value is comparable to those observed in the structures of the previously reported isomer and some other similar compounds (Hu, 2010; Tang *et al.*, 2008). In the crystal, the molecules are linked through C—H···O bonds into chains along [1 2 1] direction. The chains are connected into layers *via* C—H···π interactions (Table 1, Fig. 2).

### Experimental

To a suspension of α,α'-dibromo-*p*-xylene (1 g, 3.8 mmol) and potassium carbonate (1.05 g, 7.57 mmol) in dry acetone (25 ml), 4'-hydroxyacetophenone (1.03 g, 7.57 mmole) was added and the mixture was refluxed for 48 hr. The solvent was then evaporated under reduced pressure and the crude material was extracted with dichloromethane (3 *x* 25 ml). The combined organic layers were washed with water followed by brine and dried over anhydrous sodium sulfate. The solvent was evaporated under vacuum and the formed amorphous solid was re-crystallized from chloroform to obtain colorless crystals of the title compound (m.p. = 435–437 K).

### Refinement

Hydrogen atoms were placed at calculated positions and refined in riding mode with C—H distances of 0.95 (aryl), 0.98 (methyl) and 0.99 (methylene) Å, and *U*_iso_(H) set to 1.2 (1.5 for methyl) *U*_eq_(carrier atoms).

### Crystal data

**Table:**

C_24_H_22_O_4_	*Z* = 1
*M**_r_* = 374.42	*F*(000) = 198
Triclinic, *P*1	*D*_x_ = 1.368 Mg m^−^^3^
Hall symbol: -P 1	Mo *K*α radiation, λ = 0.71073 Å
*a* = 8.1286 (12) Å	Cell parameters from 1506 reflections
*b* = 8.1610 (7) Å	θ = 2.9–28.8°
*c* = 8.4878 (6) Å	µ = 0.09 mm^−^^1^
α = 116.164 (5)°	*T* = 100 K
β = 106.328 (7)°	Block, colorless
γ = 100.196 (7)°	0.23 × 0.19 × 0.09 mm
*V* = 454.41 (8) Å^3^	

### Data collection

**Table:**

Bruker APEXII CCD diffractometer	1654 independent reflections
Radiation source: fine-focus sealed tube	1472 reflections with *I* > 2σ(*I*)
graphite	*R*_int_ = 0.020
φ and ω scans	θ_max_ = 25.5°, θ_min_ = 2.8°
Absorption correction: multi-scan (*SADABS*; Sheldrick, 1996)	*h* = −9→9
*T*_min_ = 0.979, *T*_max_ = 0.992	*k* = −9→9
2732 measured reflections	*l* = −10→10

### Refinement

**Table:**

Refinement on *F*^2^	Primary atom site location: structure-invariant direct methods
Least-squares matrix: full	Secondary atom site location: difference Fourier map
*R*[*F*^2^ > 2σ(*F*^2^)] = 0.040	Hydrogen site location: inferred from neighbouring sites
*wR*(*F*^2^) = 0.112	H-atom parameters constrained
*S* = 1.07	*w* = 1/[σ^2^(*F*_o_^2^) + (0.0644*P*)^2^ + 0.0983*P*] where *P* = (*F*_o_^2^ + 2*F*_c_^2^)/3
1654 reflections	(Δ/σ)_max_ < 0.001
128 parameters	Δρ_max_ = 0.20 e Å^−^^3^
0 restraints	Δρ_min_ = −0.34 e Å^−^^3^

### Special details

**Table:**

Geometry. All e.s.d.'s (except the e.s.d. in the dihedral angle between two l.s. planes) are estimated using the full covariance matrix. The cell e.s.d.'s are taken into account individually in the estimation of e.s.d.'s in distances, angles and torsion angles; correlations between e.s.d.'s in cell parameters are only used when they are defined by crystal symmetry. An approximate (isotropic) treatment of cell e.s.d.'s is used for estimating e.s.d.'s involving l.s. planes.
Refinement. Refinement of *F*^2^ against ALL reflections. The weighted *R*-factor *wR* and goodness of fit *S* are based on *F*^2^, conventional *R*-factors *R* are based on *F*, with *F* set to zero for negative *F*^2^. The threshold expression of *F*^2^ > σ(*F*^2^) is used only for calculating *R*-factors(gt) *etc*. and is not relevant to the choice of reflections for refinement. *R*-factors based on *F*^2^ are statistically about twice as large as those based on *F*, and *R*- factors based on ALL data will be even larger.

### Fractional atomic coordinates and isotropic or equivalent isotropic displacement parameters (Å^2^)

**Table:**

	*x*	*y*	*z*	*U*_iso_*/*U*_eq_	
O1	1.00611 (13)	1.92652 (14)	1.27797 (14)	0.0228 (3)	
O2	0.62238 (13)	1.01503 (13)	0.83520 (13)	0.0192 (3)	
C1	1.22612 (19)	1.8825 (2)	1.4939 (2)	0.0246 (3)	
H1A	1.2907	2.0214	1.5498	0.037*	
H1B	1.3039	1.8086	1.4524	0.037*	
H1C	1.1976	1.8627	1.5904	0.037*	
C2	1.05129 (18)	1.8131 (2)	1.32364 (19)	0.0179 (3)	
C3	0.93621 (18)	1.60305 (19)	1.20855 (18)	0.0166 (3)	
C4	0.97991 (18)	1.4674 (2)	1.25479 (18)	0.0174 (3)	
H4	1.0826	1.5113	1.3695	0.021*	
C5	0.87715 (18)	1.2701 (2)	1.13753 (19)	0.0174 (3)	
H5	0.9082	1.1802	1.1722	0.021*	
C6	0.72738 (18)	1.20521 (19)	0.96773 (18)	0.0166 (3)	
C7	0.67704 (18)	1.3397 (2)	0.92338 (19)	0.0181 (3)	
H7	0.5714	1.2964	0.8115	0.022*	
C8	0.78030 (19)	1.5347 (2)	1.04150 (19)	0.0181 (3)	
H8	0.7455	1.6249	1.0095	0.022*	
C9	0.67885 (19)	0.86901 (19)	0.86356 (19)	0.0197 (3)	
H9A	0.6449	0.8585	0.9629	0.024*	
H9B	0.8132	0.9049	0.9071	0.024*	
C10	0.58484 (18)	0.67807 (19)	0.67576 (19)	0.0172 (3)	
C11	0.64548 (18)	0.64417 (19)	0.52994 (19)	0.0183 (3)	
H11	0.7453	0.7424	0.5498	0.022*	
C12	0.56143 (18)	0.4684 (2)	0.35604 (19)	0.0176 (3)	
H12	0.6038	0.4474	0.2576	0.021*	

### Atomic displacement parameters (Å^2^)

**Table:**

	*U*^11^	*U*^22^	*U*^33^	*U*^12^	*U*^13^	*U*^23^
O1	0.0258 (6)	0.0168 (5)	0.0241 (5)	0.0062 (4)	0.0077 (4)	0.0111 (4)
O2	0.0205 (5)	0.0115 (5)	0.0182 (5)	0.0045 (4)	0.0024 (4)	0.0057 (4)
C1	0.0232 (7)	0.0162 (7)	0.0236 (7)	0.0013 (6)	0.0030 (6)	0.0080 (6)
C2	0.0192 (7)	0.0161 (7)	0.0177 (7)	0.0052 (6)	0.0089 (6)	0.0079 (6)
C3	0.0171 (7)	0.0161 (7)	0.0164 (7)	0.0052 (6)	0.0080 (5)	0.0078 (6)
C4	0.0174 (7)	0.0185 (7)	0.0137 (6)	0.0064 (5)	0.0050 (5)	0.0071 (6)
C5	0.0206 (7)	0.0160 (7)	0.0178 (7)	0.0079 (6)	0.0078 (6)	0.0099 (6)
C6	0.0178 (7)	0.0143 (7)	0.0158 (7)	0.0041 (5)	0.0076 (6)	0.0064 (6)
C7	0.0181 (7)	0.0179 (7)	0.0151 (6)	0.0056 (6)	0.0041 (5)	0.0078 (6)
C8	0.0207 (7)	0.0172 (7)	0.0190 (7)	0.0080 (6)	0.0078 (6)	0.0111 (6)
C9	0.0226 (7)	0.0154 (7)	0.0190 (7)	0.0077 (6)	0.0046 (6)	0.0091 (6)
C10	0.0184 (7)	0.0143 (7)	0.0190 (7)	0.0080 (5)	0.0050 (5)	0.0094 (6)
C11	0.0168 (7)	0.0165 (7)	0.0225 (7)	0.0050 (5)	0.0060 (5)	0.0124 (6)
C12	0.0194 (7)	0.0184 (7)	0.0189 (7)	0.0089 (6)	0.0083 (5)	0.0115 (6)

### Geometric parameters (Å, °)

**Table:**

O1—C2	1.2240 (16)	C6—C7	1.3967 (19)
O2—C6	1.3617 (17)	C7—C8	1.375 (2)
O2—C9	1.4398 (15)	C7—H7	0.9500
C1—C2	1.5059 (19)	C8—H8	0.9500
C1—H1A	0.9800	C9—C10	1.5020 (18)
C1—H1B	0.9800	C9—H9A	0.9900
C1—H1C	0.9800	C9—H9B	0.9900
C2—C3	1.4866 (19)	C10—C11	1.3931 (19)
C3—C4	1.3964 (19)	C10—C12^i^	1.3954 (19)
C3—C8	1.4035 (19)	C11—C12	1.3864 (19)
C4—C5	1.387 (2)	C11—H11	0.9500
C4—H4	0.9500	C12—C10^i^	1.3954 (19)
C5—C6	1.3972 (19)	C12—H12	0.9500
C5—H5	0.9500		
			
C6—O2—C9	117.64 (10)	C8—C7—C6	120.06 (12)
C2—C1—H1A	109.5	C8—C7—H7	120.0
C2—C1—H1B	109.5	C6—C7—H7	120.0
H1A—C1—H1B	109.5	C7—C8—C3	121.19 (13)
C2—C1—H1C	109.5	C7—C8—H8	119.4
H1A—C1—H1C	109.5	C3—C8—H8	119.4
H1B—C1—H1C	109.5	O2—C9—C10	108.13 (10)
O1—C2—C3	120.37 (12)	O2—C9—H9A	110.1
O1—C2—C1	120.78 (12)	C10—C9—H9A	110.1
C3—C2—C1	118.84 (12)	O2—C9—H9B	110.1
C4—C3—C8	117.90 (13)	C10—C9—H9B	110.1
C4—C3—C2	122.90 (12)	H9A—C9—H9B	108.4
C8—C3—C2	119.15 (12)	C11—C10—C12^i^	118.81 (13)
C5—C4—C3	121.68 (12)	C11—C10—C9	119.68 (13)
C5—C4—H4	119.2	C12^i^—C10—C9	121.51 (12)
C3—C4—H4	119.2	C12—C11—C10	120.58 (13)
C4—C5—C6	119.14 (13)	C12—C11—H11	119.7
C4—C5—H5	120.4	C10—C11—H11	119.7
C6—C5—H5	120.4	C11—C12—C10^i^	120.60 (12)
O2—C6—C7	115.22 (12)	C11—C12—H12	119.7
O2—C6—C5	124.87 (12)	C10^i^—C12—H12	119.7
C7—C6—C5	119.91 (13)		

Symmetry codes: (i) −*x*+1, −*y*+1, −*z*+1.

### Hydrogen-bond geometry (Å, °)

**Table:**

Cg1 is the centroid of the C3–C8 ring.

**Table:**

*D*—H···*A*	*D*—H	H···*A*	*D*···*A*	*D*—H···*A*
C11—H11···O1^ii^	0.95	2.54	3.4362 (18)	158.
C12—H12···Cg1^iii^	0.95	2.61	3.5078 (17)	158

Symmetry codes: (ii) −*x*+2, −*y*+3, −*z*+2; (iii) *x*, *y*−1, *z*−1.
